# TURNING THE OXYGEN DIAL AS A THERAPY: HYPOXIA TREATMENT FOR MITOCHONDRIAL DYSFUNCTION

**DOI:** 10.1093/geroni/igz038.1460

**Published:** 2019-11-08

**Authors:** Isha Jain

**Affiliations:** University of California, San Francisco, San Francisco, California, United States

## Abstract

Mitochondrial disease affects 1 in 4800 live births, with little in the way of therapies. We found that chronic hypoxia extends the life of a Complex 1 disease model by 5-fold. Starting hypoxia therapy at a late-stage of disease can even reverse the MRI-detectable lesions. At the other extreme, mild hyperoxia greatly exacerbates disease and leads to death within several days. These findings have now led to a phase 1 clinical trial in healthy volunteers, with the ultimate goal of human translation. We believe we have identified a new mode of treatment that will be broadly applicable to different forms of mitochondrial dysfunction, ranging from rare inborn errors of metabolism to more common, age-associated pathologies. We believe that “turning the oxygen dial” to low or high oxygen will serve as a novel therapeutic for a range of conditions in the coming years.

